# Sequence based analysis of U-2973, a cell line established from a double-hit B-cell lymphoma with concurrent *MYC* and *BCL2* rearrangements

**DOI:** 10.1186/1756-0500-5-648

**Published:** 2012-11-22

**Authors:** Sean D Hooper, Xiang Jiao, Elisabeth Sundström, Farah L Rehman, Christian Tellgren-Roth, Tobias Sjöblom, Lucia Cavelier

**Affiliations:** 1 Department of Immunology, Genetics and Pathology, Rudbeck Laboratory, Uppsala University, Uppsala, 751 85, Sweden; 2 The Breakthrough Breast Cancer Research Centre, Institute of Cancer Research, Mary-Jean Mitchell Green Building, Chester Beatty Laboratories, Fulham Road, London, SW3 6JB, UK

**Keywords:** Double hit lymphoma, Sequencing, Chromosomal rearrangements

## Abstract

**Background:**

Double-hit lymphoma is a complex and highly aggressive sub-type of B-cell lymphoma, which has recently been classified and is an area of active research interest due to the poor prognosis for patients with this disease. It is characterized by the presence of both an activating *MYC* chromosomal translocation and a simultaneous additional oncogenic translocation, often of the *BCL2* gene. Recently, a cell line was established from a patient with this complex lymphoma and analyzed using conventional tools revealing it contains both *MYC* and *BCL2* translocation events.

**Findings:**

In this work, we reanalyzed the genome of the cell line using next generation whole genome sequencing technology in order to catalogue translocations, insertions and deletions which may contribute to the pathology of this lymphoma type.

**Conclusions:**

We describe the cell line in much greater detail, and pinpoint the exact locations of the chromosomal breakpoints. We also find several rearrangements within cancer-associated genes, which were not found using conventional tools, suggesting that high throughput sequencing may reveal novel targets for therapy, which could be used concurrently with existing treatments.

## Findings

Rearrangements in the human genome are a common feature of B-cell lymphomas with recurrent reciprocal chromosomal translocations present in approximately 40% of cases [1]. Certain translocations are considered specific for certain types of lymphoma. For example, t(8;14)(q24;q32) or variant translocations leading to constitutive activation of the *MYC* transcription factor are a hallmark of Burkitt's lymphoma and are present in 80-95% of cases [2]. Furthermore, in follicular lymphomas, a characteristic translocation occurs between chromosomes 14 and 18 (t(14;18)) which deregulates the apoptosis-inhibiting gene *BCL2*[3], resulting in a failure to respond to cell death signals. In rare cases, these rearrangements are concurrent or sequential, and these cases are therefore sometimes referred to as Double Hit (DH) lymphoma. According to the most recent WHO guidelines these tumors are classified as “B cell lymphoma unclassifiable with features intermediate between DLBCL and BL [1]. Several cases of DH lymphoma have been described "(e.g. [4-6]) and have attracted research interest due to their unique cytogenetic features and relatively poor prognosis. Recently a stable B-cell line (U-2973) was established [7] from a DH lymphoma patient. This derived cell line does not necessarily represent the full clonality or tumorigenesis of the patient, but retains the main translocations initially identified in the patient. U-2973 was analyzed using conventional cytogenetic tools such as fluorescent in situ hybridization (FISH), G-band staining and multicolor FISH. These approaches showed concurrent translocations at t(8;12)(q24;p12) and t(14;18)(q32;q21). However, the primary drawback of these methods is the low level of detail and the breakpoints of translocations can rarely be determined within tens or hundreds of kilobases. To address this issue, we have repeated the analysis of U-2973 with massive parallel sequencing on the SOLiD (ABI) platform. In short, DNA from U-2973 was sheared and fragments of on average 1kbp and 2.5kbp were selected for sequencing. By sequencing both ends of DNA fragments, we can capture not only translocations but also deletions and insertions with a high level of resolution. As a result we report a detailed map of chromosomal regions with extensive rearrangements not previously observed using conventional methods. We also pinpoint the DNA sequence at the breakpoint of t(8;12) and describe a transposition of DNA from chromosome 20 to chromosome 8 which could not be detected using conventional tools.

### Conventional analysis

The M-FISH and karyotyping were performed as the DNA was extracted for the sequence analysis to verify the karyotype reported earlier (Additional file 1: Figure S1A and Additional file 2: Figure S2A) [7]. FISH hybridizations using gene specific probes (*MYC* and *IGH-BCL*2) and whole chromosome paint hybridizations (WCP8 and WCP12; WCP 17 and WCP20) were performed to confirm the findings of the M-FISH, on cells from an independent culture. (Additional file 1: Figure S1B-D and Additional file 2: Figure S2B). The analysis showed basically the same karyotype as reported earlier with some discrepancies, probably due to changes during cell culture namely the reported trisomy 13, which we did not detect and a rearranged chromosome 20, that contained extra material from chromosome 17, which was earlier reported as containing material from chromosome 19. All cells analyzed showed the same karyotype in two independent cultures indicating the presence of a single clone in the sample used for DNA extraction and relative stability of the cell line. However we cannot confirm that the cell line faithfully represents all the lymphoma cells in the patient at diagnosis, but it rather constitutes a single clone selected when establishing the cell line in culture. The karyotype was further verified by SNP6 microarray analysis, which did not detect any trisomy 13 and showed additional 17-q material probably corresponding to the fragment incorporated into chromosome 20.

### Detailed rearrangements

Deletions are characterized by a loss of DNA on one or both alleles. Through sequencing, we discovered deletions in U-2973 not previously described. We find for instance exonic deletions in the potential oncogene *PIGU*[8,9], and intronic deletions in *PRKCA*, which is overexpressed in some Burkitt's lymphomas [10]. Regional intergenic deletions include 6.8kbp on chromosome 5, 4.5kbp on chromosome 1, and 7.2kbp on chromosome 8. For DNA gains, the major insertions are roughly 800bp into *ANO3* on chromosome 11 and the protocadherin *PCDH9* on chromosome 13. We also find two cases of inversions involving exonic material in *NKAIN3* and *TSPAN8*, a cell surface antigen, which were found to be overexpressed in some human tumor cell lines [11].

**Figure 1:**
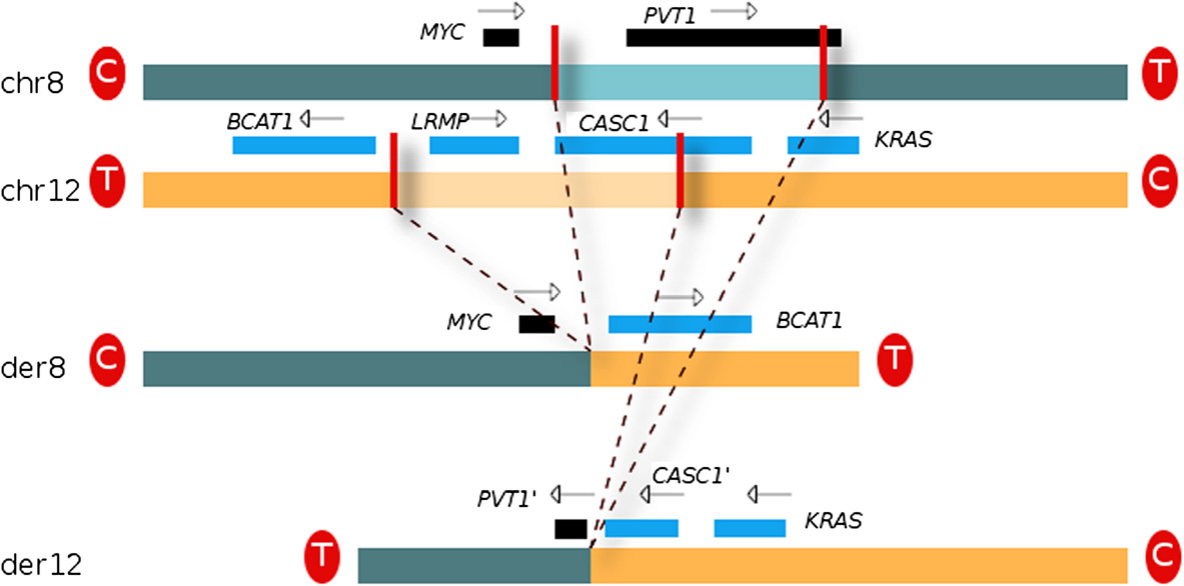
**Detail of the t(8:12) translocations as validated by sequencing.** This translocation has resulted in a possible heterozygous partial loss of *CASC1*, and has an unknown effect on *MYC*. *LRMP* has also been lost from the derivative chromosome 12. C and T denotes the direction of the centromere and telomere.

The characteristic rearrangements of a DH lymphoma are the two chromosomal translocations involving *MYC, BCL2 och IGH*. The translocation breakpoints (Figure 1) on the derivative chromosome 8 der(8)t(8;12) were sequenced using Sanger sequencing and located to nucleotide coordinate 128835920 on chr8; 13kbp downstream of *MYC* and 45kbp upstream of *PVT1*, and at position 25082917 on chr12; roughly 12kbp upstream of *LRMP*, which appears to be deleted as a consequence of the translocation. The reciprocal translocation der(12) was not sequenced, but is positioned at roughly 129189kbp on chr8 and at approximately 25189kbp on chr12. Thus, it appears that most of the *CASC1* gene has been lost, although one copy is likely to remain on the third copy of chromosome 12 as is also the case for *LRMP*. Most of the sequence of *PVT1* has been lost from the genome, since there are no remaining copies of the original chromosome 8.

The t(14;18) translocation is also validated by both libraries and PCR, and subsequently Sanger sequenced. One breakpoint lies within the IGH gene on chr14 and the other roughly 20kbp upstream of *BCL2* on chr18. This is consistent with previous observations of *IGH-BCL2* fusions in 96% of the blood sample [7] and in the FISH-analysis on the cell line (Additional file 1: Figure S1C). Furthermore, the breakpoint between chr14 and chr18 is interspersed with a 31 bp sequence which has no homology to the reference (hg18) genome. This sequence may have been extant in the genome before the translocation, but is more likely to have been inserted as a consequence of a non-homologous end joining of a double stranded chromosomal break.

In the previous analysis [7], it was established that the biallelic t(8;12) translocation involved *MYC* on chr8 and potentially the *LRMP* gene on chr12, but more detailed positions could not be determined. In this study, the biallelic translocation could be more accurately determined and the positions of the breakpoints suggest that *LRMP* may have been lost on the two derivative chromosomes 12. However, potential regulatory elements of *LRMP* may remain after the loss of sequence, and may deregulate other genes. There are no previously identified promoter regions or regulatory elements in this region, but it is interesting to note that the mouse ortholog to *LRMP* extends upstream to this chromosomal location. Considering that *LRMP* is known to be expressed in germinal centre B-cells and in diffuse large B-cell Lymphomas of the “germinal” center subtype [12], we hypothesize that regulatory elements originally located upstream of the *LRMP* gene and juxtaposed to the *MYC*-locus, might be involved in the de-regulation of *MYC*. Interestingly, a recent report shows a case of a primary B-cell lymphoma with a t(8;12) translocation also involving *MYC* and *LRMP*[13]. Although the exact position of the breakpoint at *LRMP* was not determined, FISH analysis showed that the 5’ region of *LRMP* was translocated to the *MYC* locus also in this case. Thus it might be of interest to determine the recurrence of *MYC/LRMP* rearrangements in a larger number of B-cell lymphomas.

Furthermore, the sequence analysis strongly suggests a translocation between chromosome 8 and 20. This translocation is supported by a large number of reads such as the previous translocations. However no t(8:20) translocation could be observed by either FISH or karyotyping (see Additional file 2: Figure S2B). Since sequence reads could rather represent an insertion of DNA from one chromosome into the other, without otherwise disrupting chromosome 8 or 20, we designed primers considering the two possible insertion structures with a segment of 8 on 20 and vice versa. The PCR reactions thereafter confirmed that roughly 5 kbp of intronic DNA within the *VPS13B* gene on chromosome 8 was inserted into chromosome 20 at position 58001229 and replacing some extant DNA including intronic sequences and exon 10 of the *CDH6*gene (Additional file 3: Figure S3). Whether or not this insertion has a driving role is unclear, but this case illustrates the sensitivity of mate-pair sequencing and also the need for a rigorous interpretation of the results.

Besides the three sequence-verified major translocation events, we also find other events with lower sequence read support and therefore rather represent transpositions of small regions of DNA, repetitive sequences or subclonal populations. The full list of translocations and affected genes is provided as Supplementary Material. This may be useful in identifying possible recurrent aberrations when compared to other lymphomas, and could also be informative when working with the U-2973 cell line. Thus comparisons to sequences from other lymphomas might clarify which of these events are recurrent and of pathogenic importance.

### Methods

The U-2973 cell line was established from a biopsy with the informed consent of the patient and approved by the local ethical review board (Uppsala 00–275) as described in the original study [7]. DNA was prepared and sequenced on the ABI SOLiD parallel sequencing system according to the manufacturer's instructions. We prepared and sequenced two mate-pair libraries; one with an insert size of 2.5kbps, and one with an insert size of 1.5kbps. Thereafter, both ends of the fragment were ligated to adaptors. Using the Corona Lite software package (ABI), the 25bp reads were aligned to the human reference genome (NCBI build 36) and reads with unique matches on both ends were retained, resulting in a total of 119M read-pairs.

## Competing interests

The authors declare no competing interests.

## Author contributions

LC and SH conceived and designed the study. LC coordinated experiments and analysis. SH and CTR performed the bioinformatics analysis. LC performed the M-FISH analysis. All authors participated in discussions of different parts of the study. SH and ES wrote the manuscript. All authors read and approved the manuscript.

## Supplementary Material

Additional file 1 **Figure S1A.** G-band karyotype from the cell line retrieved from the double-hit cell lymphoma. 47,XY,der(8)t(8;12)(q24.21;p12.1)*x*2,+12,der(12)t(8;12)(q24.21; p12.1)*x*2,t(14;18)(q32;q21),der(20)t(17;20)(q23;q13) chromosomes were harvested using a routine bone marrow protocol with one hour colcemide treatment. The slides were subsequently stained using standard Trypsin-Giemsa and karyotyped using the Cytovision software. S1B-D, FISH hybridization of chromosomes retrieved from the double-hit cell lymfoma. Chromosomes were harvested as above and hybridized with the MYC BA probe, the IGH-BCL2 DF probe (Abbott) and the WCP17 and WCP20 (Metasystems), according to the manufacturer’s instructions. The pictures were retrieved using a Zeiss microscope and analyzed using the ISIS software. Click here for file

Additional file 2 **Figure S2.** Multicolor FISH and WCP hybridizations. S2A. M-FISH hybridization showing the t(8;12), t(14;18) and the t(17;20) translocations. S2B. WCP of chromosome 8 and 12, showing the two derived chromosomes 8, the two derived chromosomes 12 and a normal chromosome 12. Click here for file

Additional file 3 **Figure S3.** Detail of an insertion of extragenous DNA and deletion of material on chromosome 20. This rearrangement was predicted by sequencing and undetected by conventional methods, but yet confirmed by PCR. Click here for file
