# Supplementary figures and images for: Sequence based analysis of U-2973, a cell line established from a double-hit B-cell lymphoma with concurrent MYC and BCL2 rearrangements
Source: BMC Res Notes. 2012 Nov 22;5:648. doi: 10.1186/1756-0500-5-648 (PMC3534606; doi:10.1186/1756-0500-5-648)

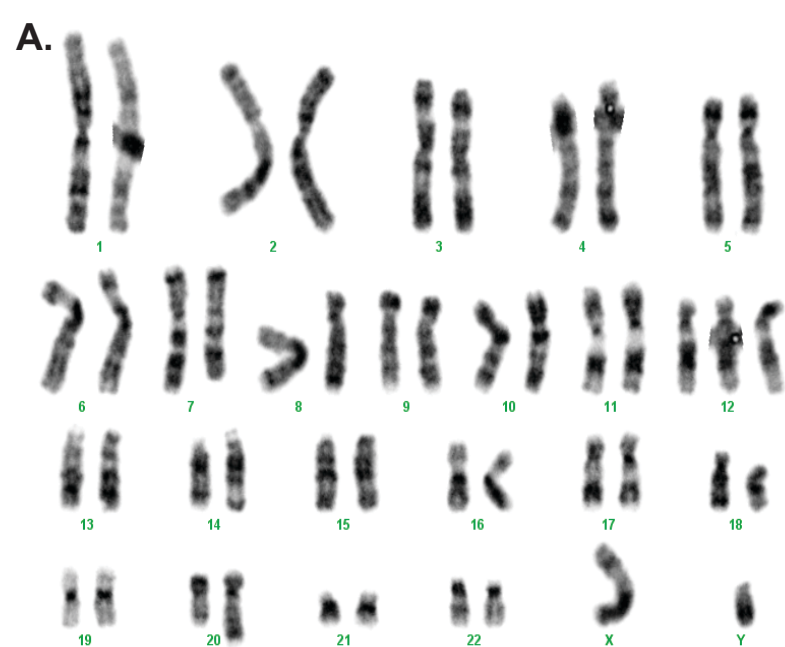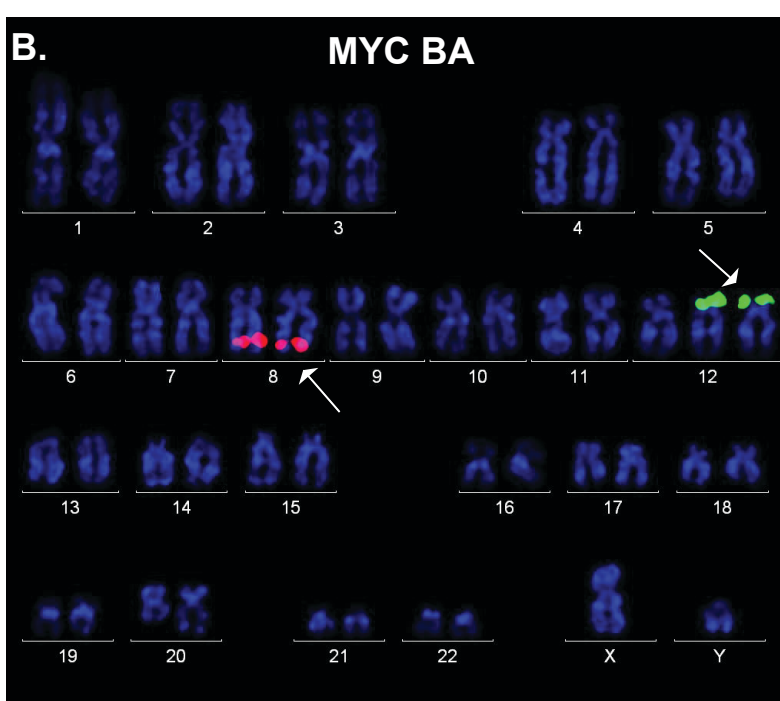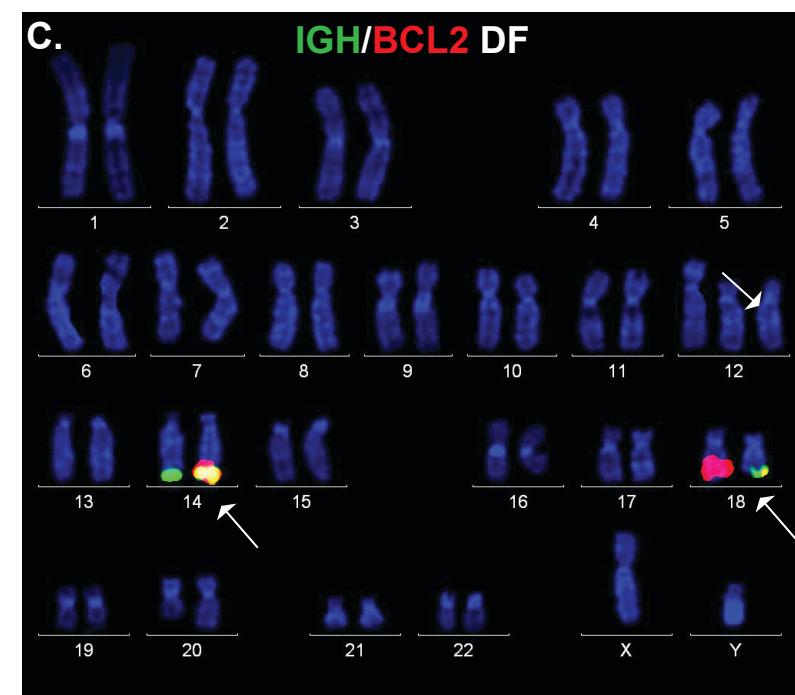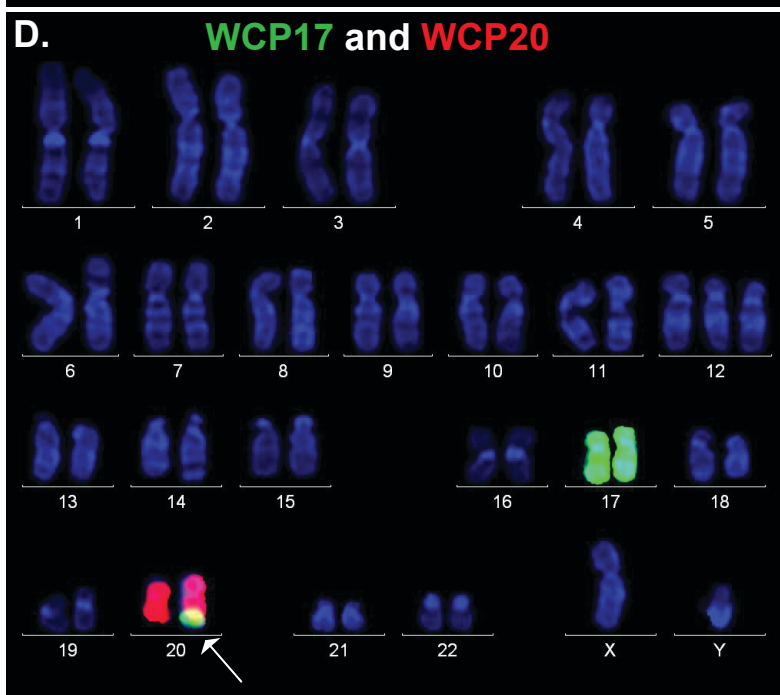

Supplement: Additional file 1 — Figure S1A. G-band karyotype from the cell line retrieved from the double-hit cell lymphoma. 47,XY,der(8)t(8;12)(q24.21;p12.1)x2,+12,der(12)t(8;12)(q24.21; p12.1)x2,t(14;18)(q32;q21),der(20)t(17;20)(q23;q13) chromosomes were harvested using a routine bone marrow protocol with one hour colcemide treatment. The slides were subsequently stained using standard Trypsin-Giemsa and karyotyped using the Cytovision software. S1B-D, FISH hybridization of chromosomes retrieved from the double-hit cell lymfoma. Chromosomes were harvested as above and hybridized with the MYC BA probe, the IGH-BCL2 DF probe (Abbott) and the WCP17 and WCP20 (Metasystems), according to the manufacturer’s instructions. The pictures were retrieved using a Zeiss microscope and analyzed using the ISIS software. [file 1756-0500-5-648-S1.pdf]

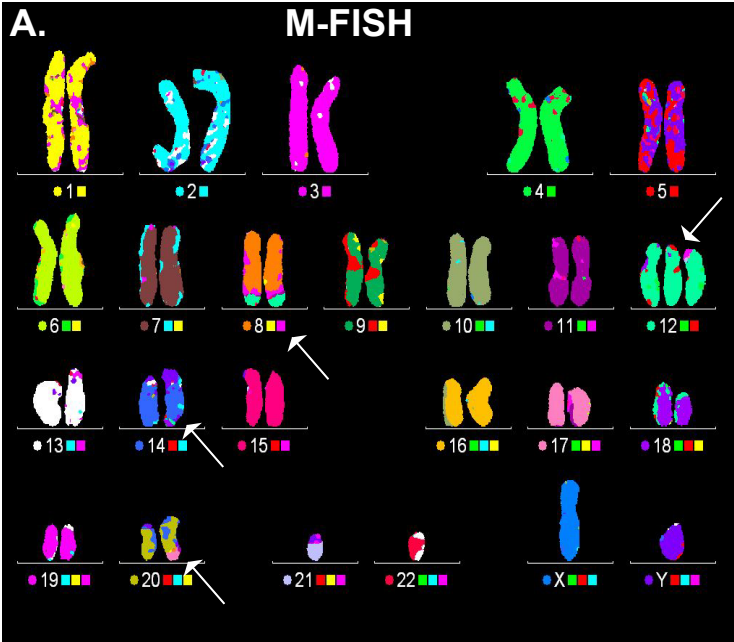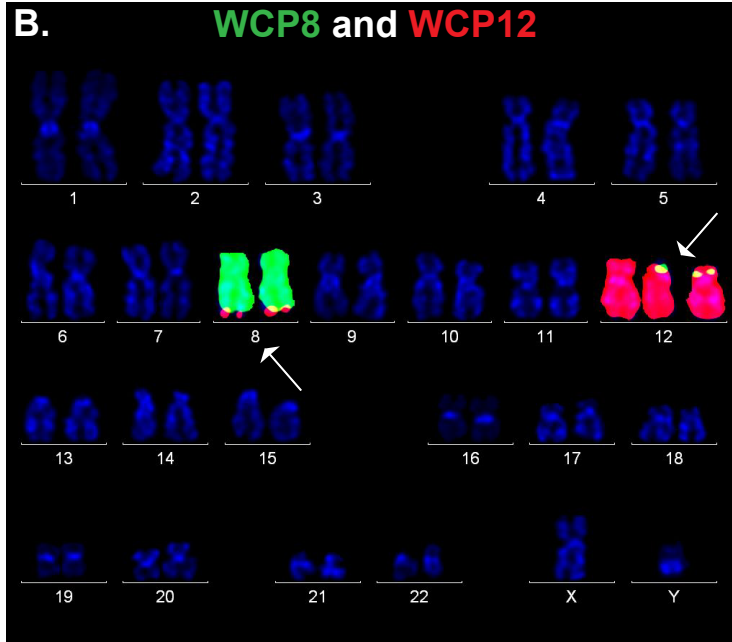

Supplement: Additional file 2 — Figure S2. Multicolor FISH and WCP hybridizations. S2A. M-FISH hybridization showing the t(8;12), t(14;18) and the t(17;20) translocations. S2B. WCP of chromosome 8 and 12, showing the two derived chromosomes 8, the two derived chromosomes 12 and a normal chromosome 12. [file 1756-0500-5-648-S2.pdf]

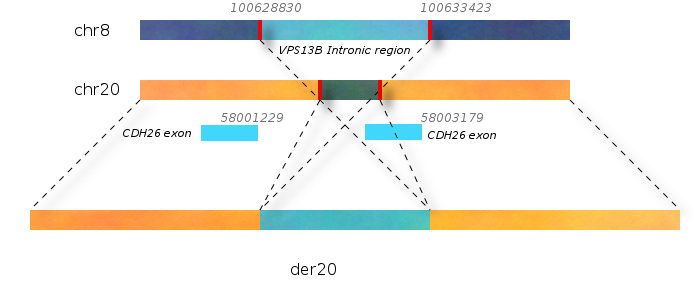

Supplement: Additional file 3 — Figure S3. Detail of an insertion of extragenous DNA and deletion of material on chromosome 20. This rearrangement was predicted by sequencing and undetected by conventional methods, but yet confirmed by PCR. [file 1756-0500-5-648-S3.png]
